# Dendritic cell nediated inhibition of lentiviral infection

**DOI:** 10.1186/1742-4690-9-S2-P178

**Published:** 2012-09-13

**Authors:** A Drake, E Browne, R Phennicie, J Chen

**Affiliations:** 1 MIT Koch Institute for Integrative Cancer Research, Cambridge, MA, USA

## Background

Lentiviral entry to quiescent lymphocytes represents a 'time bomb' waiting for cellular activation to spread infection. In order to undergo immune activation T cells interact with dendritic cells presenting peptide:MHC complexes 'sampling' them to look for agonist peptides and receiving survival signals from self peptides. This makes the dendritic cell:T cell interaction an ideal checkpoint to contain lentiviral infection of quiescent lypmhocytes.

## Methods

We have used replication defective lentiviral vectors expressing reporter genes and/or HIV proteins to study the innate immune response to lentiviral infection in vitro using primary mouse and human cells and in vivo in B6 mice. We have used flow cytometry and PCR to quantify the infection rates in various culture and adoptive transfer conditions to identify conditions where successful viral infection is inhibited.

## Results

Activating T cells exposed to dendritic cells display inhibited lentiviral vector infection compared to activated control cells. This infection reduction is mediated by dendritic cell:T cell contact. Dendritic cell mediated inhibition of infection reduces over time as cells become fully activated and proliferate. When using lentiviral vectors similar results are seen in primary human T cells in culture. However when replication defective vectors expressing HIV proteins are used to infect T cells de novo expression of HIV proteins in the host cell prevents DC mediated inhibition. We have gone on to use knockout mice to assess the role of established innate immune sensing pathways in DC mediated inhibition but have not yet identified the molecular sensor mediating this phenomenon.

## Conclusion

The inhibition of productive T cell infection by lentiviral vectors caused by contact with dendritic cells represents a previously undescribed innate immune response which we have called DC mediated inhibition. The successful lentiviral pathogen HIV overcomes DC mediated inhibition enhancing its replication and obscuring this immune response.

